# The activation of cGAS-STING pathway offers novel therapeutic opportunities in cancers

**DOI:** 10.3389/fimmu.2025.1579832

**Published:** 2025-06-09

**Authors:** Yumin Wang, Yonglin Zhu, Yuwei Cao, Yulin Li, Zhe Zhang, Joshua S. Fleishman, Sihang Cheng, Jichao Chen, Mingchao Ding

**Affiliations:** ^1^ Department of Respiratory and Critical Care Medicine, Aerospace Center Hospital, Peking University Aerospace School of Clinical Medicine, Beijing, China; ^2^ Department of Pharmaceutical Sciences, College of Pharmacy and Health Sciences, St. John’s University, New York, NY, United States; ^3^ Department of Radiology, Peking Union Medical College Hospital, Chinese Academy of Medical Sciences, Beijing, China; ^4^ Department of Peripheral Vascular Intervention, Aerospace Center Hospital, Peking University Aerospace School of Clinical Medicine, Beijing, China

**Keywords:** CGAS, STING, agonist, cancers contents, small-molecule compounds

## Abstract

The cyclic GMP-AMP synthase (cGAS)/stimulator of interferon genes (STING) pathway are crucial elements of the type I interferon (type I IFN) response. cGAS senses both exogenous and endogenous DNA within cells, labeling cGAS-STING as a pivotal anti-tumor immunity mechanism, autoimmunity, sterile inflammatory responses, and cellular senescence. The cGAS-STING pathway, a pivotal innate immune axis, modulates tumorigenesis via diverse effector responses. Emerging evidence have shown that activating of cGAS-STING pathway functions as a therapy to kill cancers. Insights into the biology of the cGAS-STING pathway have enabled the discovery of small-molecule agents which have the potential to activate cGAS-STING axis in cancers. In this review, we first outline the principal components of the cGAS-STING signaling cascade. Then we explore recent advancements in understanding the cGAS-STING signaling pathway, with particular emphasis on its activation mechanisms and roles in tumor cancer killing. Next, we summarize a list of bioactive small-molecule compounds which activate the cGAS-STING axis, reviewing their potential applications. Finally, we discuss key limitations of this new proposed therapeutic approach and provide possible techniques to overcome them. This review highlights a novel groundbreaking therapeutic possibilities through activating cGAS-STING in cancers.

## 1 Introduction

Cyclic GMP/AMP (cGAMP) synthase (cGAS), along with the endoplasmic reticulum (ER)-associated stimulator of interferon genes (STING), are crucial elements of the innate immune response (1, 2). Microbial DNA is a pathogen-associated molecular pattern (PAMP), the main ‘‘molecular threat’’ needed to activate the DNA sensing protein cGAS. cGAS promotes the synthesis of the cyclic dinucleotide cGAMP which binds to STING, initiating trafficking and migration from the ER to the Golgi, where it recruits TANK-binding kinase 1 (TBK1) and the transcription factor interferon regulatory factor 3 (IRF3). Phosphorylated IRF3 dimerizes and translocates into the nucleus, enhancing the expression of type I interferons (IFN-I) and IFN-stimulated genes (ISGs) (2–4). Increasing evidence reveals that the over-activation and aberrant regulation of the cGAS-STING axis triggers undesired outcomes such as neuroinflammation and neurodegeneration, contributing to neurological disorders and accelerating disease progression (1–3, 5–8).

In the past decade, interest has increased in elucidating the role of cGAS-STING in cancers. cGAS-STING pathway modulators are new and attractive targets for targeted medicine against cancers. Unlike chemotherapy, radiotherapy, or checkpoint inhibitors (e.g., PD-1/PD-L1 blockers) that primarily modulate adaptive immunity or directly kill cells, cGAS-STING agonists broadly activate the innate immune system (9–11). This triggers dendritic cell maturation, cross-presentation of tumor antigens, and recruitment of diverse immune cells, fostering a more systemic and sustained anti-tumor response. It can convert immunologically “cold” tumors (T cell-poor) into “hot” tumors (T cell-inflamed), addressing a limitation of many conventional therapies. cGAS-STING activation enhances tumor immunogenicity and primes the tumor microenvironment for checkpoint inhibitors. By promoting antigen presentation and cytokine production (e.g., type I interferons), it amplifies T cell responses that checkpoint inhibitors rely on, overcoming resistance to monotherapy (12–19). Given the essential role of cGAS-STING signaling in the pathogenesis of cancers, drug discovery targeting the cGAS-STING axis has expanded rapidly (20, 21).

In this review, we first outline the principal components of the cGAS-STING signaling cascade. From such we discuss recent research that highlights general mechanisms by which cGAS-STING contributes to cancers. Then, we summarize a list of bioactive small-molecule compounds which modulate the cGAS-STING axis, reviewing their potential clinical applications. Finally, we discuss key limitations of this new proposed therapeutic approach and provide possible techniques to overcome them.

## 2 cGAS-STING pathway

**Figure 1:**
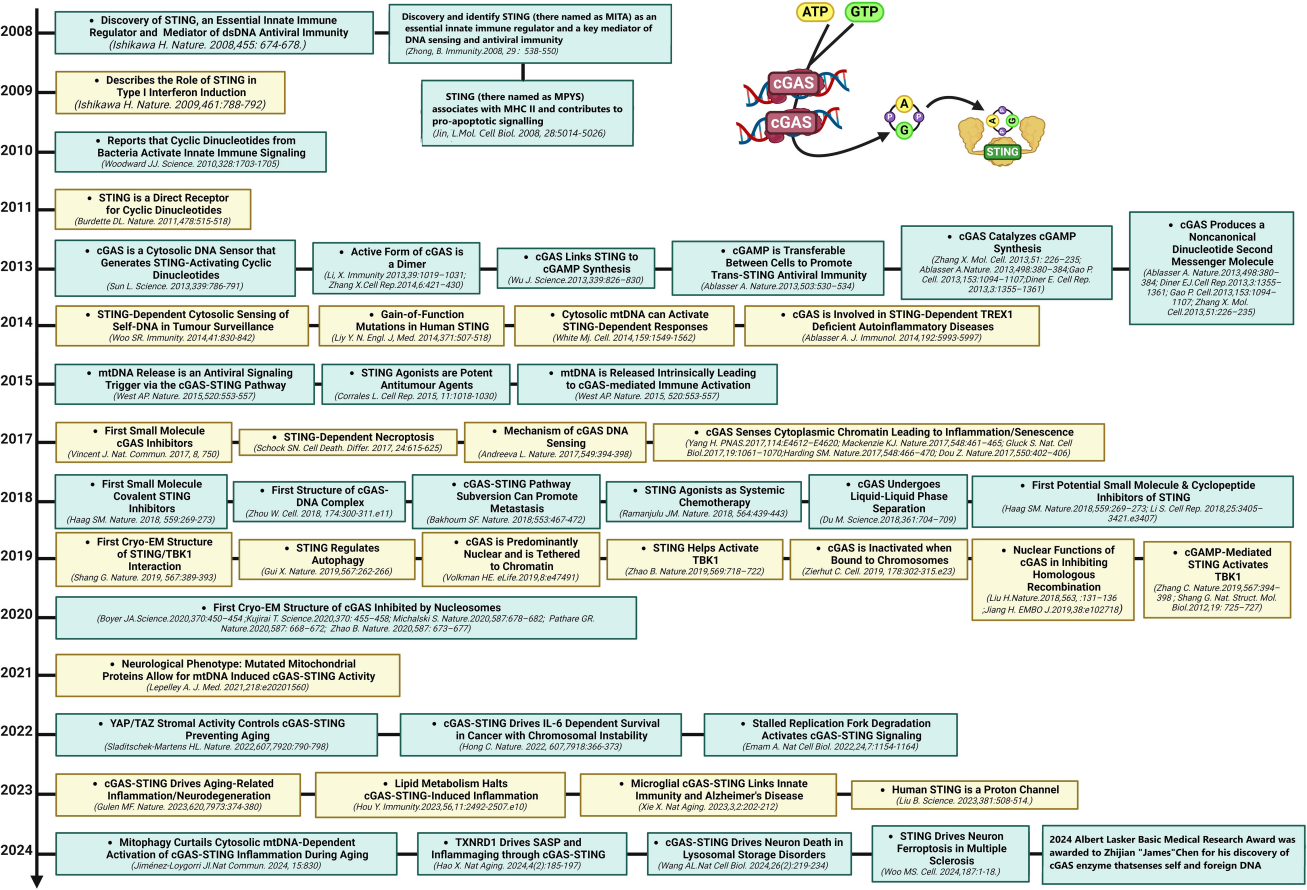
Milestones in cGAS-STING research. Timeline depicting the scientific discoveries of the cGAS-STING pathway from 2008 to 2024.

**Figure 2:**
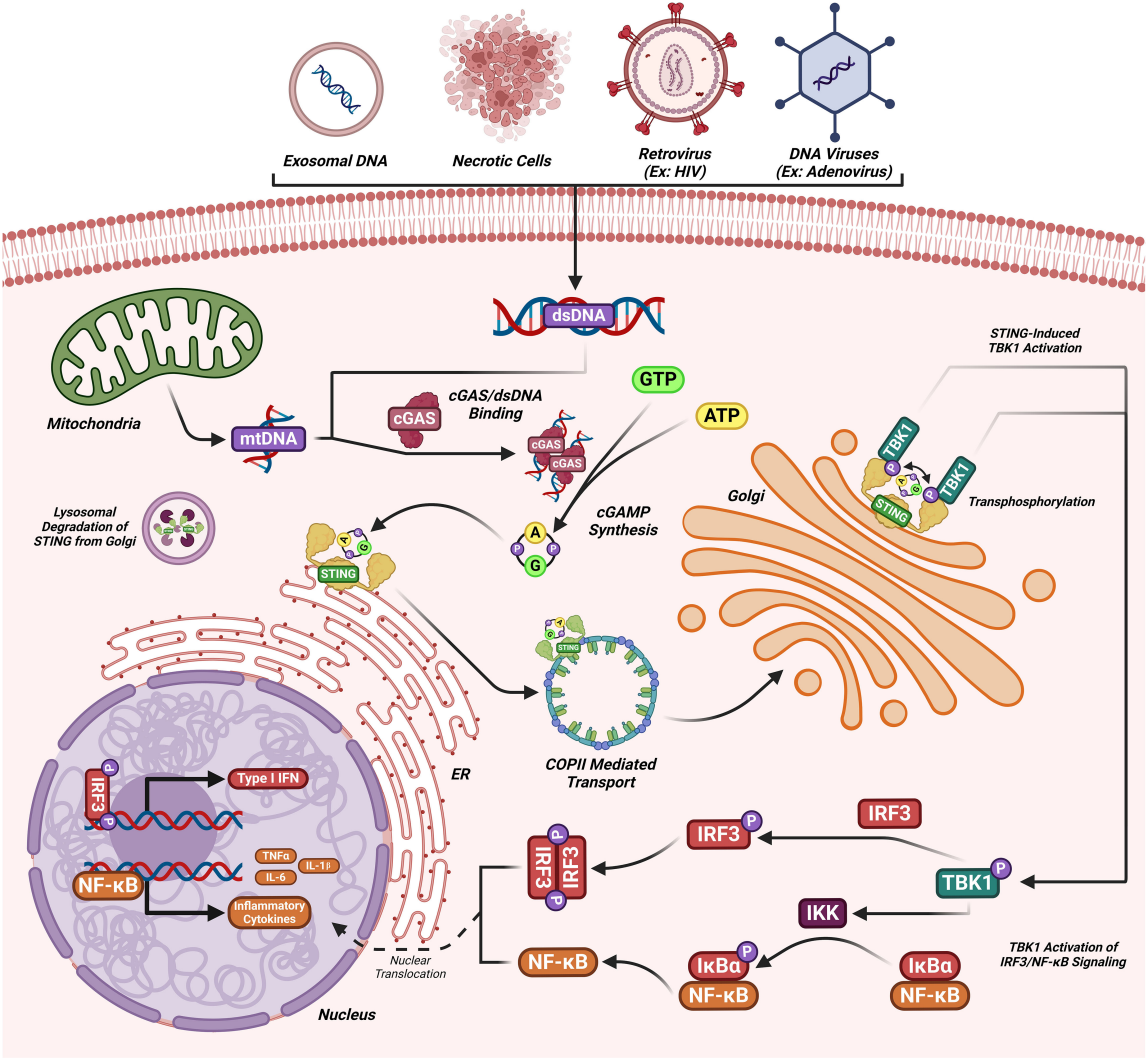
The cGAS-STING signaling cascade. dsDNA (introduced by viral or extracellular origins) binds to cGAS catalyzing the synthesis of cGAMP. cGAMP upon binding to STING transports to the Golgi where TBK1 transphosphorylation occurs. Phosphorylated TBK1 can then phosphorylate IRF3 inducing the nuclear transcription of IFN 1 genes or can activate IKK, inducing NF-kB mediated cytokine synthesis. STING is then recycled from the Golgi apparatus and degraded.

The DNA-sensing nucleotidyl transferase enzyme cyclic GMP/AMP (cGAMP) synthase (cGAS) is upstream of STING (22, 23). Key developments in cGAS-STING research is shown in **Figure 1**. Timeline depicting the scientific discoveries of the cGAS-STING pathway The cGAS-STING signaling axis detects pathogenic extranuclear DNA and initiates a type I interferon innate immune activation, physiologically used against microbial infections, making cGAS-STING an integral component the innate immune response (24). Belonging to a member of the nucleotidyl transferase (NTase) enzyme family, cGAS is also known as MB21D1 (24). STING is otherwise known as endoplasmic reticulum interferon stimulator (ERIS) (25), N-terminal methionine-proline-tyrosine-serine plasma membrane transpanner (MPYS) (26, 27), mediator of interferon regulatory factor 3 (IRF3) activation (MITA) (28) or transmembrane protein 173 (TMEM173) (24). The DNA sensor cGAS senses microbial (i.e. viral, bacterial, protozoal) double-stranded DNA (dsDNA), independent of sequence. cGAS may be activated by either endogenous DNA, mitochondrially-released DNA, or genotoxic stress-mediated extranuclear chromatin, placing cGAS-STING as a crucial signaling axis in autoimmunity, the sterile inflammatory response, and the induction of cellular senescence (24). An overview of the cGAS-STING signaling axis is illustrated in **Figure 2**. In mammalian cells, cGAS induces the synthesis of the secondary-messenger cyclic GMP/AMP (cGAMP), forming a crucial cytosolic DNA-sensing mechanism. cGAS binding to dsDNA induces a conformational change, activating it and initiating enzymatic activity (29–33). Active cGAS catalyzes and converts guanosine triphosphate (GTP) and adenosine triphosphate (ATP) into 2′,3′-cyclic GMP-AMP (cGAMP) (23). Subsequently, cGAMP binds to and activates STING, a ~40-kDa endoplasmic reticulum (ER)-localized transmembrane protein adaptor (23, 34, 35), to form homomeric quaternary ensembles of varying stoichiometry (36, 37). After activation, STING translocates from the ER to the Golgi, where it recruits TANK binding kinase 1 (TBK1) and IκB kinase (IKK), which respectively phosphorylate interferon regulatory factor 3 (IRF3) and the nuclear factor-κB (NF-κB) inhibitor IκBα (24). TBK1 transphosphorylates itself, the C-terminal domains of STING, and subsequently IRF3 (24). Meanwhile, STING engages and activates IKK to trigger NF-κB signaling (24), which works together with a robust IFN response to orchestrate the immunologically-driven clearance of intracellular bacteria, retroviruses, and DNA viruses (24). IRF3 dimerizes and translocates to enter the nucleus, transcriptionally activating genes which encode type I interferons, such as interferon-β (IFNβ), initiating antiviral defense mechanisms (24). The phosphorylation of IκBα leads to the nuclear translocation of NF-κB, enhancing the expression of proinflammatory cytokines such as tumor necrosis factor (TNF) and IL-6 (3). STING is trafficked to endolysosomes for degradation after activation (24). cGAS and STING are tightly regulated by transcriptional, posttranslational, and protein degradation mechanisms, for which we refer readers to a specific review for further discussion (24). cGAS senses cytosolic dsDNA in response to tissue injury or pathogenic invasion, which allows for the cGAS-STING axis to regulate various cellular functions, such as protein synthesis, IFN/cytokine production, autophagy, senescence, metabolism, and specific mechanisms of cell death (24). The cGAS-STING axis is vital for tissue homeostasis and host defense, while dysfunction of cGAS-STING activates pro-inflammatory signaling pathways, leading to inflammatory, autoimmune, degenerative diseases, and cancer (24).

## 3 cGAS-STING in cancers

**Figure 3:**
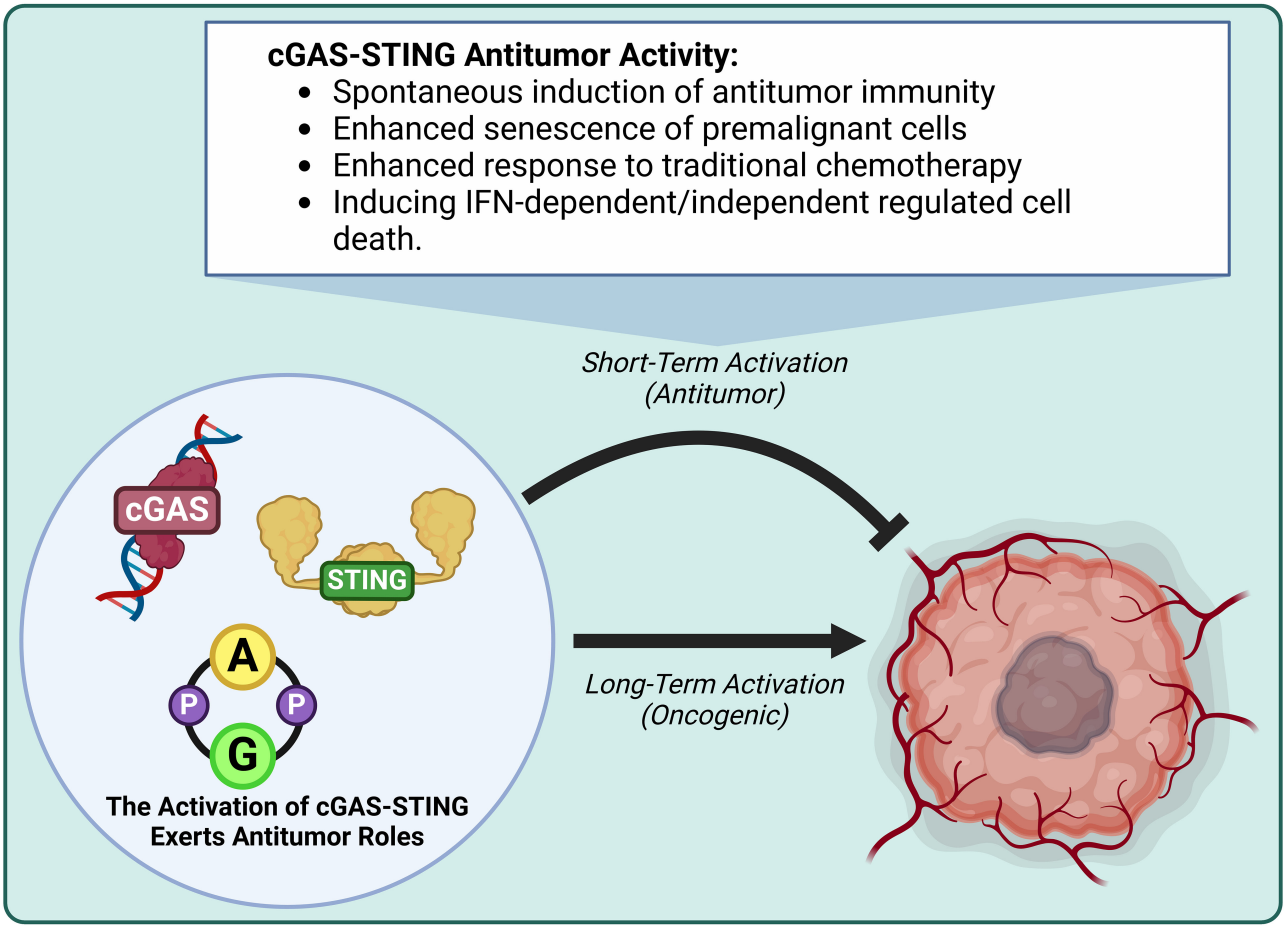
The activation of cGAS-STING exerts antitumor activity. Short-term cGAS-STING activity is antitumor, while long-term activity is oncogenic. Antitumor activity induces antitumor immunity, enhances premalignant cell senescence, enhances responsiveness to traditional chemotherapy, and induces IFN-dependent/independent regulated cell death.

**Figure 4:**
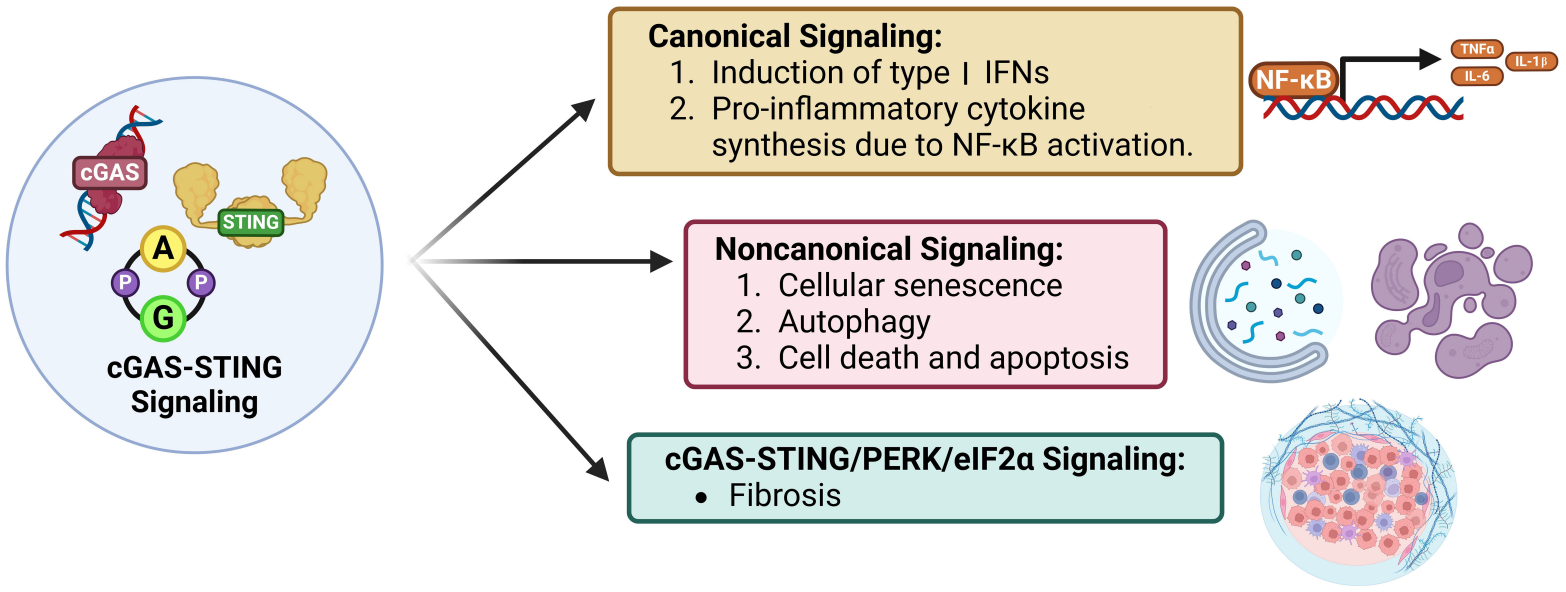
Signaling modes of cGAS-STING. Canonical cGAS-STING signaling induces type I IFN expression and an NF-kB-mediated pro-inflammatory response. STING dependent, cGAS, IFN, and TBK1 independent non-canonical signaling induces cellular senescence, autophagy, and apoptosis. Lastly, cGAS-STING/PERK/eIF2a signaling induces intra-organ fibrosis.

New evidence reveals that the cGAS-STING axis is crucial in cancer development (38), impacting all aspects of tumorigenesis from initial malignancy to metastasis (39). The cGAS-STING axis is a well-known double-edged sword, in which acute activation promotes antitumor effects and chronic inflammation promotes oncogenic growth/metastasis (40–42). In this upcoming section we will discussion how endogenous oncogenic processes are further modified by cGAS-STING axis activity. We refer readers to a recent excellent review for detailed discussion of the detrimental outcomes of cGAS-STING in cancer (40, 41, 43). The activation of cGAS-STING exerts an antitumor role by inducing spontaneous antitumor immunity, enhancing senescence in premalignant cells, responding to classic cancer therapies, and inducing regulated cell death via IFN-dependent and IFN-independent pathways (41, 44)(**Figures 3**, **4**).

### 3.1 Activation of cGAS-STING induces regulated cell death

**Figure 5:**
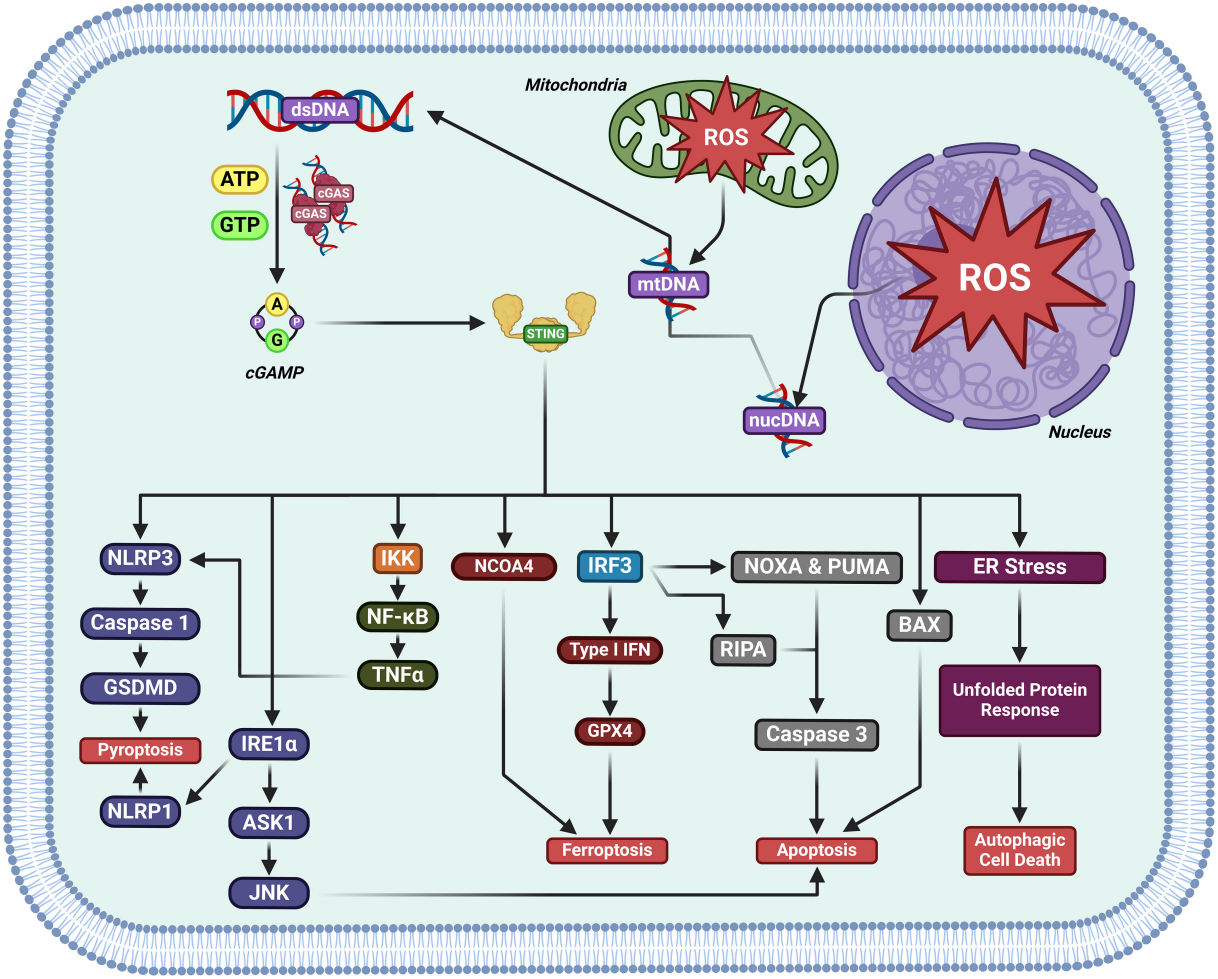
The activation of cGAS-STING induces regulated cell death in cancer. cGAS-STING pathway activation has been documented to kill cancer cells by either inducing pyroptosis, ferroptosis, apoptosis, or autophagic cell death.

Studies have categorized cell death mechanisms as ‘uncontrolled necrosis’ and ‘regulated cell death (RCD)’, which contains ‘regulated necrosis (or non-apoptotic RCD)’ and apoptosis (45–48). Non-apoptotic RCD consists of pyroptosis, ferroptosis, cuproptosis, disulfidptosis, and autophagic cell death,et.al (49, 50). Activation of cGAS-STING induces RCD (51) (**Figure 5**).

#### 3.1.1 Apoptosis

STING activity induces apoptosis. STING agonists have been found to induce apoptosis in B and T cells *in vitro*/vivo (52–54). STING/IRF3/p53 axis activity upregulates Noxa and Puma, directly promoting apoptosis (55).

#### 3.1.2 Autophagy

cGAS-STING and autophagy have been shown to be interplayed, which may influence the progression of cancer. STING mediates autophagy.cGAS-STING pathway can trigger autophagy in several ways in innate immunity (reviewed in ref (56, 57)).

#### 3.1.3 Ferroptosis

The nuclear cysteine protease cathepsin B (CTSB) triggers DNA damage and cGAS-STING1 activation to induce autophagydependent ferroptosis by degrading GPX4, thereby facilitating the anticancer activity of sorafenib in PDAC (58). STING promotes ferroptosis in pancreatic cancer by promoting MFN1/2-dependent mitochondrial fusion. Erastin, the pro-ferroptotic inducer enhances STING accumulation of in the mitochondria, where it interacts with MFN1/2 and promotes mitochondrial fusion, enhancing ROS production and lipid peroxidation. STING or MFN1/2 knockout reduces the sensitivity of pancreatic cancer samples to ferroptosis *in vitro* xenograft mice. Cumulatively, STING promotes ferroptosis via MFN1/2-dependent mitochondrial fusion (59). cGAS-STING axis activity promoted by manganese enhances mitochondrial lipid peroxidation and ROS production by upregulating IFN-1 release, directly preventing DHODH activity and therefore inducing ferroptosis in tumors (60).

#### 3.1.4 Pyroptosis

Radium-223 inhibits tumor progression through triggering pyroptosis. DNA damage from 223 Ra promotes STING/NLRP3 axis activity, resulting in pyroptosis, dendritic cell, and T cell maturation (61).

### 3.2 Activation of cGAS-STING induces cancer-immunity cycle

**Figure 6:**
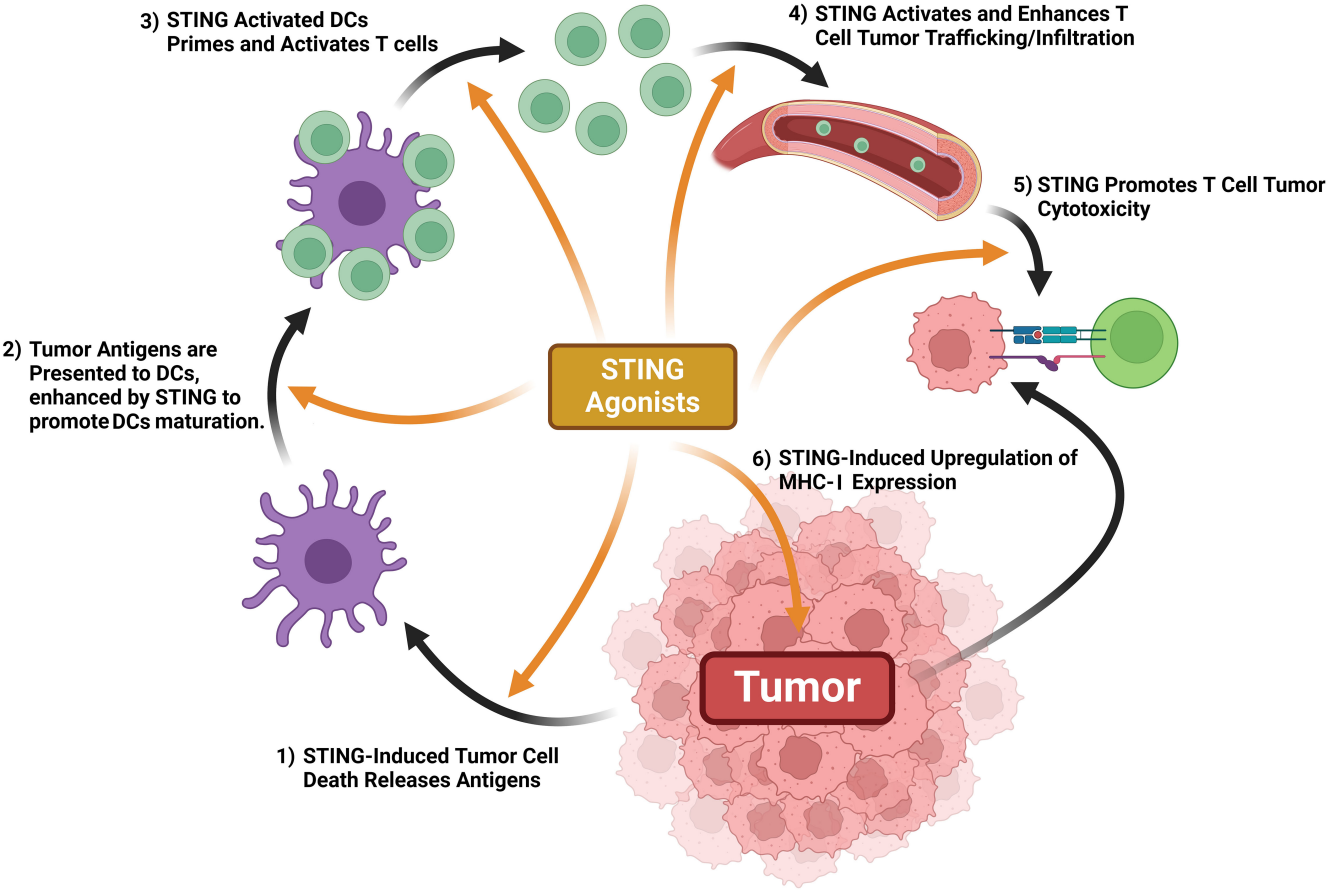
STING activation promotes antitumor immune mechanisms. STING activation promotes tumor antigen release, DC maturation once exposed to tumor antigens, T cell priming, T cell trafficking & tumor infiltration, tumor expression of MHC-1, and T cell-mediated cytotoxicity.

The cancer-immunity cycle (CI cycle) provides a framework to understand what events promote an anticancer immunological response (62). Antigen-presenting cells (APCs), phagocytose cancer antigens and display them to T cells, which activates effector T cells to infiltrate the tumor, activate cytotoxic T cells, and kill tumor cells. In turn, newly killed cancer cells release more cancer-specific antigens which further promotes immunologically-driven cancer targeting (62, 63). This cycle teaches that T cell activity is promoted by a series of steps, some of which being extrinsic to the immune system and the cancer (63). cGAS-STING axis activity promotes every step of T cell immune defenses against cancer, effectively turning an immunologically “cold” tumor into a “hot” tumor being targeted by multiple immune responses (**Figure 6**) (64). cGAS-STING axis activity directly promotes tumor cell death. STING activity within DCs induces IFN-1 secretion and promotes maturation, whereas STING activity in T cells enhances their priming, activation, and chemokine output. STING activity enhances normalization of tumor blood vessels, allowing for easier T cell infiltration and upregulates MHC Class I expression, enhancing T cell-tumor recognition (reviewed in ref (43, 64)).

### 3.3 Activation of cGAS-STING enhances senescence in premalignant cells

Senescence is a stress-inducible state of terminal cell cycle arrest and complex proinflammatory secretions, including chemokines, proteases, cytokines, and is also referred to as the senescence-associated secretory phenotype (SASP) (65). Senescence is a hallmark of both cancer/aging (66, 67). The SASP facilitates growth inhibition in a paracrine manner (68). SASP-associated chemokines can recruit immune cells to remove cells which contain damaged DNA (69). Senescence is normally associated with telomere shortening or the steady-state accumulation of DNA damage (70). Exogenous stresses such as ROS or ionizing radiation may also induce senescence, including that of classical chemotherapy and targeted therapy (65). Inducing cellular senescence may function as a defense mechanism against oncogenesis and metastasis. From such, it may be currently used as a common mechanism underlying current anticancer therapies (65, 71–73). Together, senescence, and more importantly the mechanisms underlying its induction hold much importance in the development of novel anticancer therapies (71–73).

New studies have displayed that the DNA sensing functionality of the cGAS-STING axis directly promotes senescence and the SASP (74–77). Decreased senescence was observed cGAS or STING knockout cells after irradiation, oncogene expression, serial passage, or treatment with DNA-damaging drugs, all of these are known to create micronuclei and activate cGAS-senescence (74–77). cGAS or STING knockout prevented the SASP, where impaired clearance of RasV12-expressing cells tumorigenesis (74, 75). STING knockout increased tumor susceptibility in colitis-related mice cancer models (78, 79), inevitably indicating that the SASP-promoted cGAS-STING axis prevents tumorigenesis by reinforcing senescence or enhancing the immune-targeted clearance of aberrant cells. Decreased cGAS or STING expression was observed in several cancer cell lines (22, 80). Lower cGAS or STING expression in cancer samples was correlated to worse patient outcomes with hepatocellular carcinoma and lung adenocarcinoma, suggesting the importance of functional cGAS-STING axis activity in proper tumor suppression (77, 81).

### 3.4 Ubiquitination-mediated inhibition of cGAS-STING in cancers

Emerging research has revealed multiple E3 ubiquitin ligases as critical regulators of the cGAS-STING pathway in cancer biology. Recent studies by Fu et al. (82) and Li et al. (83) demonstrated distinct mechanisms through which different TRIM family members modulate this pathway. TRIM41 was identified as a negative regulator that interacts with IDI1, a key enzyme in the mevalonate pathway, to promote cGAS ubiquitination and subsequent degradation, effectively dampening cGAS-STING signaling (82). Meanwhile, TRIM21 exerts its suppressive effect through mitochondrial regulation, enhancing VDAC2 ubiquitination to prevent mtDNA release via inhibition of VDAC2 oligomerization, thereby blocking radiation-induced STING activation and antitumor immunity (83). Concurrently, Liu et al. uncovered the paradoxical role of ARIH1 in cancer immunotherapy resistance (84). Their work revealed that ARIH1 deficiency promotes ICB resistance by disrupting the DNA-PKcs-STING signaling axis, while cisplatin-treated ICB-insensitive tumors showed compensatory ARIH1 upregulation. Mechanistically, ARIH1 overexpression facilitates DNA-PKcs ubiquitination and degradation, thereby activating STING signaling to enhance cytotoxic T-cell infiltration and synergize with PD-L1 blockade (84). This STING-mediated immunostimulatory effect was specifically dependent on non-phosphorylated cGAS, as demonstrated by the abolished response in cells expressing the phosphomimetic T68E/S213D mutant (84). Collectively, these findings highlight the therapeutic potential of targeting E3 ligase networks to modulate cGAS-STING pathway activity. TRIM29 restricts antiviral innate immunity against DNA virus infections by targeting STING for degradation (85). Recently, TRIM29 was also shown to promote viral myocarditis by enhancing ROS-mediated TBK1 oxidation and inhibition (86). Additionally, TRIM29 deficiency has been shown to control viral enteritis by regulating inflammasome activation (87). Furthermore, TRIM18 knockout has been demonstrated to control viral myocarditis and organ inflammation through the upregulation of TBK1-mediated antiviral immunity (88). Given the critical roles of TRIM29 and TRIM18 in controlling cGAS-STING pathway and cancer development, it is deserved to investigate the role of TRIM29 and TRIM18 in controlling cGAS-STING pathway in cancers.

## 4 Therapeutic potential of cGAS-STING activation in cancers

**Figure 7:**
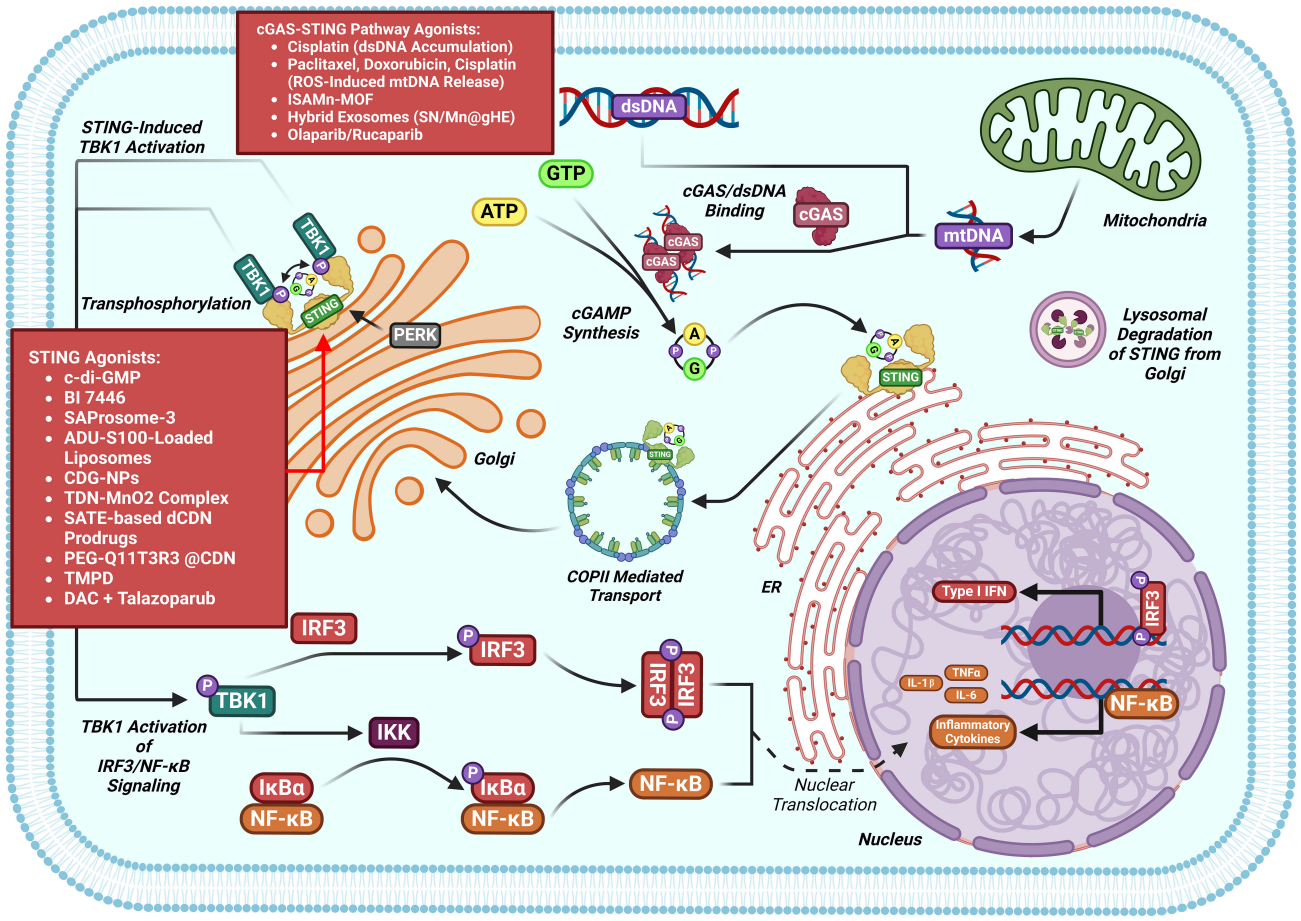
cGAS-STING pathway modulators in the setting of cancers. Multiple classes of cGAS-STING antagonists have shown benefit against cancers models.

Suppression of cGAS-STING was found in various human malignancies, leading to growing interest in small-molecule agonists that reactivate this pathway to kill cancer (89, 90). Several compounds have already demonstrated therapeutic potential by targeting cGAS-STING in cancers (**Figure 7**). A summary of compounds functions as cGAS-STING agonists that include STING agonist, Poly(ADP-ribose) polymerase inhibitors (PARPi), nanodrug, chemotherapy agent and others drugs are itemized in **Table 1**.

**Table 1:** Emerging compounds activating cGAS-STING for cancer treatment.

Cancers	Compounds	Compounds type	Experimental models	Findings	Ref
Breast cancer	c-di-GMP	STING agonist	4T1 cells -bearing mouse	↓4T1 cell growth in mice; ↑IFN-β level and phosphorylation of STING, TBK1, IRF3 and STAT1 in tumor mass of tumor-bearing mice	(91)
Breast cancer	BI 7446	STING agonist	4T1 BALB/c mouse tumor model	↑Tumor regression; ↑long-term immunologic memory against autologous tumor rechallenge.	(92)
Breast cancer	SAProsomes-3	STING agonist	Orthotopic 4T1 breast tumor model	↑Secretion of inflammatory cytokines;↑ tumoricidal immune landscape; ↑durable remission of established tumors and postsurgical tumor-free survival; ↓metastatic burden;↓tumor metastasis and recurrence after surgery.	(93)
Breast cancer	ADU-S100-loaded liposomes	STING agonist	THP-1 Dual cells	↑STING signaling; ↑TNFα and IFNβ production;↑Maturation of Bone Marrow-Derived Dendritic Cells (BMDCs); ↑ maturation of dendritic cells.	(94)
Cervical cancer	diABZI, SR-717 and MSA-2	STING agonist	Caski cells bearing NOD-SCID mice	↓Tumor growth of HPV-infected cervical cancer;	(95)
CRC	SHR1032	STING agonist	MC38 tumor-bearing C57BL/6 mice	↑cGAS-STING signaling; ↓tumor growth; ↑IFNβ, IL-6 and TNFα in both tumors and plasma.	(96)
CRC	SAProsomes-3	STING agonist	MC38 cells tumor -bearing female BALB/c mice	↑Secretion of inflammatory cytokines; ↑tumoricidal immune landscape; ↓durable remission of established tumors and postsurgical tumor-free survival;↓metastatic burden; ↑tumor regression; prolongs survival rate.	(93)
CRC	diBSP01	STING agonist	Zebrafish xenograft model bearing MC38 cells	↓Tumor growth.	(97)
CRC	BSP16	STING agonist	MC38 tumor-bearing C57BL/6 mice	↓Tumor regression and durable antitumor immunity; ↑ STING signaling.	(98)
CRC	DMXAA	STING agonist	Luciferase-MC38 cells were injected into C57BL/6 mice	↓Liver metastases; ↑survival of mice;↓ALT and AST in serum samples; ↑TBK1 and IRF3.	(99)
CRC	ALG-031048	STING agonist	CT26 mouse colon carcinoma model	↓Tumor growth.	(100)
HCC	rgFlu/PD-L1	–	COS-I, MDCK, MIHA, SMMC-7721, HepG2, MHCC-97L, and Huh-7	↑Killing of HCC;↓PD-L1 expression; ↑apoptosis in HepG2 cells;↓viability and function of CD8+ T cells; ↑activating the cGAS-STING pathway.	(101)
HCC	ROD peptide hydrogel	STING agonist		↑Percentages of DCs, CD4+ T cells and CD8+ T cells; ↓lowest level of Treg cells; ↑expression levels of IFN-γ and TNF-α; ↑ expression levels of pSTING, pIRF3, and IFN-β; ↑ surviving mice.	(102)
HCC	cyclic dinucleotide	STING agonist	C57BL/6/DEN	↓Tumor size.	(103)
Gastric cancer	Metformin	cGAS-STING agonist	BGC823, AGS, and SGC7901	↑ Activation of the cGAS-STING signaling pathway; ↓SOX2 and AKT phosphorylation.	(104)
KP soft tissue sarcoma	DMXAA	STING agonist	Pleomorphic sarcoma -bearing C57Bl/6 mice	↓Tumor growth;↑long-term survival;↓ tumor metastasis.	(105)
Acute myeloid leukemia	SHR1032	STING agonist	MOLM-16 cells; MV4-11 cells	↓AML cell growth;↑apoptosis in AML cells.	(96)
Glioblastoma	CL656	STING agonist	GBM stem cell line 005 into the striatum of the mouse	↓Tumor volume and diameter;↑ survival of mice.	(106)
Multiple cancer	M335	STING agonist	C57BL/6J or Balb/c mice bearing MC38, CT26, or B16F10 cells	↑ Activation of TBK1-IRF3-IFN axis in a STING-dependent; ↑immune cells; ↓tumor burden.	(107)
Breast cancer	Vinylphosphonate-CDNs		4T1 tumor-bearing mice	↓Tumor growth.	(108)
NSCLC	Olaparib and rucaparib	PARPi		↑ Cytoplasmic chromatin fragments with characteristics of micronuclei; ↑ activation of cGAS-STING, downstream type I IFN signaling, and CCL5 secretion.	(109)
TNBC	Olaparib and rucaparib	PARPi		Produces cytoplasmic chromatin fragments with characteristics of micronuclei; ↑activates cGAS-STING;↓ type I IFN signaling, and CCL5 secretion.	(109)
TNBC	PMZH nanoplatform	PARPi	4T1 tumor-bearing mice	↓Tumor growth; ↑STING signaling activation; ↑DC maturation; ↓ lung metastasis	(110)
ccRCC	DAC+talazoparib	DNA HMA/PARPi	SETD2-deficient ccRCC	↑ Cytotoxicity *in vitro* in SETD2-deficient ccRCC cell lines;↑apoptosis; ↑ DNA damage; ↓insufficient DNA damage repair;↑genomic instability;↑immune responses;↑STING;↑ viral mimicry by activating transposable elements; ↓ growth of SETD2-deficient ccRCC in *in vivo*.	(111)
TNBC	TMPD	Nanodrug	4T1 tumor-bearing mice	↓Tumor growth;↑STING signaling activation;↑immunogenic cell death (ICD);↑ DC maturation and CD8+ T cell infiltration;↑antitumor effects.	(112)
Breast cancer/CRC	LND-CDN	Nanodrug	Orthotopic 4T1 breast cancer/MC38 tumors	↓Tumor regression; ↑robust T-cell activation	(113)
Breast cancer	SN/Mn@gHE		4T1 tumor -bearing BALB/c mice	↑TAMs polarization to the M1 phenotype; ↑cGAS-STING activation; ↑DCs maturation;↑CTL infiltration and NK cell recruitment to the tumor region;↑anti-tumor and antimetastatic efficacy.	(114)
CRC	PEG-Q11T3R4@CDN	Nanodrug	CT26 cells tumor -bearing female BALB/c mice	↓Tumor growth; ↑survival rate; ↓ tumor metastasis;↑ activating CD8 + T cells to target distant tumors.	(115)
CRC	SATE-based prodrugs of dCDNs	Nanodrug	CT26-Luc tumor -bearing BALB/c mice	↑STING pathway; ↓ CT26-Luc tumor growth; ↓established tumor.	(116)
HCC	TDN-MnO2 complex	Nanodrug	Hepa1-6 cells tumor -bearing C57BL/6J(B6) mice	↑Macrophage activation; ↑anti-tumor response.	(117)
Melanoma	Eu-GAMP-NPs	Nanodrug	B16F10-OVA tumor-bearing mice	↓Tumor growth; ↑the survival of tumor-bearing mice in both therapeutic and protective models	(118)
Melanoma	ISAMn-MOF	Nanodrug	B16F10 tumor-bearing mice	↓Tumor growth and metastasis; ↑mouse survival; ↑cGAS-STING Pathway	(119)
Melanoma	CDG-NPs	Nanodrug	melanoma-bearing mice	↑ Retention and intracellular delivery of CDG in the tumor site; ↑STING activation and TME immunogenicity; ↑STING-mediated anti-tumor immunity.	(120)
Multiple cancer	STING-activating nanoparticles	Nanodrug	Multiple cancer	↑Vascular normalization; ↑infiltration, proliferation, and function of antitumor T cells; ↑response to immune checkpoint inhibitors and adoptive T cell therapy.	(121)
Multiple cancer	Hydrated- prenylated xanthone			↑STING; ↑production of pro-inflammatory cytokines.	(122)
Bladder cancer	Cisplatin	Chemotherapy agent	T24 and TCCSUP cell lines; MB49 cells tumor-bearing C57BL/6 mice	↑Immune response; ↑cGAS-STING signal; ↓proliferation of bladder cancer; ↑ infiltration percentages of CD8+ T cells and dendritic cells in mice; ↑ accumulation of dsDNA and the release of chromatin.	(123)
Breast cancer	Paclitaxel, doxorubicin, and cisplatin	Chemotherapy agent	E0771-OVA-Zs Green tumor-bearing C57BL/6J mice	↑CD8+ T anti-tumor immunity through stimulating cancer cell-autonomous type I interferon induction via ROS-triggered oxidized mtDNA sensing by cGAS-STING.	(94)
PDAC	GB1275	CD11b agonists	Orthotopically implanted in wild type and CD11b-null mice	↑Activates CD11b;↓PDAC progression;↓TAM infiltration, and increases CD8+ T cell number and proliferation; ↑activated CD11b on TAMs to drive anti-tumor immunity and restrain tumor growth;↑STING in TAMs	(124)
Gastric cancer	Anlotinib	TKIs		↓PD-L1 expression; ↑cGAS-STING/IFN-β pathway;↓gastric cancer cells proliferation, migration, and immune escape.	(125)
NSCLC	Cetuximab plus avelumab	anti-PD-L1 drug/an anti-EGFR drug	NSCLC patient-derived ex vivo 3D spheroid cultures	↑Cancer cell growth inhibition; ↑activates NK cell;↑gene expression of CCL5 and CXCL10, two STING downstream effector cytokines, and of IFNβ.	(126)
Breast cancer/melanoma	Podofilox	Microtubule destabilizer	B16F10 or 4T1 cell tumor -bearing C57BL/6 mice	↑cGAMP-mediated immune response; ↑STING oligomerization and activation; ↓ trafficking-mediated STING degradation; combination of cGAMP and podofilox had profound antitumor effects on mice by activating the immune response through host STING signaling.	(127)
Melanoma	CPs-CDN	Chimeric polymersomes	B16F10 melanoma -bearing mice	↑Activation of STING in the tumor microenvironment and tumor draining lymph nodes, giving significantly better tumor repression.	(128)

ccRCC, clear-cell renal cell carcinomas; CDN,2’, 3’-cGAMP, a cGAS-STING pathway agonist; lyOK-432, hydrogel loaded with lysed OK-432; ROD, ROD was an injectable hydrogel for effectively loading lyOK-432 and doxorubicin; *PARPi, Poly(ADP-ribose) polymerase inhibitors; TMPD, doxorubicin (DOX)-loaded PEG-PLGA nanoparticles and further coated with manganese (Mn2+)-tannic acid (TA) networks. MSA-2, non-nucleotide STING agonist; SAProsome, STING agonist pro-drug liposome; SHR1032, a novel small molecule non-CDN STING agonist; S-acylthioalkyl ester (SATE)-based prodrugs of deoxyribose cyclic dinucleotides (dCDNs); TDNs, tetrahedral DNA nanostructures; DAC, DNA hypomethylating agents 5-aza-20 -dexoxydytidine; the PARPi talazoparib (BMN-673); ↑, increase OR promote; ↓, decrease OR suppress.

### 4.1 STING agonist

c-di-GMP inhibits 4T1 cell growth and increases phosphorylation of STING, TBK1, IRF3 and STAT1 and the IFN-β level in tumor-bearing mice (91). BI 7446, a potent cyclic dinucleotide STING agonist, produces a durable and potent tumor inhibition and a long-term immunologic memory against autologous tumor rechallenge (92). SAProsomes-3 decreases metastatic burden and elicits durable remission of established tumors through stimulating secretion of inflammatory cytokines and creates a tumoricidal immune landscape (93). SAProsomes-3 promotes postsurgical tumor-free survival and decreases tumor metastasis and recurrence after surgery (93). ADU-S100-loaded liposomes facilitates the maturation of Bone Marrow-Derived Dendritic Cells (BMDCs) and promotes the maturation of dendritic cells through activating STING signaling to enhance TNFα and IFNβ production (94).

The STING agonists SHR1032 (96), SAProsomes-3 (93), diBSP01 (97), BSP16 (98), DMXAA (99), and ALG-031048 (100) all inhibit tumor growth through inhibiting activation of STING in colorectal cancer. The injectable hydrogel loaded with doxorubicin (DOX) and lysed OK-432 (lyOK-432) promotes an antitumor immunity through activating the STING pathway, conferring effective therapy for residual hepatocellular carcinoma (HCC) after incomplete radiofrequency ablation (102). The cyclic dinucleotide 3′3′-cAIMP reduces tumor size in DEN-induced C57BL/6 HCC model (103). Metformin functions as a cGAS-STING agonist to promote immunotherapy through activating the cGAS-STING signaling pathway by blocking SOX2 and AKT phosphorylation in gastric cancer (104).

### 4.2 PARPi

Poly (ADP-ribose) polymerase inhibitors (PARPi) that targets poly (ADP-ribose) polymerase are currently approved to treat a range of tumor types harboring defects of genes contribute to homologous repair (HR), including BRCA1 and BRCA2 (129). Olaparib and rucaparib produces cytoplasmic chromatin fragments with characteristics of micronuclei, which activate cGAS-STING and downstream type I IFN signaling to enhance CCL5 secretion in NSCLCL and TNBC (109). The combination of DNA hypomethylating agents 5-aza-2’-dexoxydytidine (DAC) with BMN-673 (the PARPi talazoparib) increases cytotoxicity in SETD2-deficient ccRCC cell lines (111). DAC and talazoparib induces apoptotic, increases genomic instability, DNA damage, and insufficient DNA damage repair. DAC and talazoparib elevates immune responses, upregulates STING, and enhances viral mimicry through activating transposable elements (111). DAC and talazoparib suppresses the growth of SETD2-deficient ccRCC *in vivo* (111).

### 4.3 Nanodrugs

The manganese-phenolic network platform (TMPD) inhibits tumor growth and elicits strong antitumor effects 4T1 tumor-bearing mice through promoting STING signaling activation and promotes DC maturation and CD8+ T cell infiltration, thus (112). LND-CDN that conjugates STING-activating cyclic dinucleotides (CDNs) to PEGylated lipids via a cleavable linker and incorporated them into lipid nanodiscs (LNDs), elicits tumor regression and facilitates robust T-cell activation in breast cancer and CRC (113). The SN/Mn@gHE, multifunctional hybrid exosomes that fuses genetically engineered exosomes carrying tumor cells-derived CD47 with M1 macrophages-derived exosomes, which are further encapsulated with DNA-targeting agent (SN38) and STING-agonist (MnO_2_). SN/Mn@gHE have tumor-targeting capacity and induce TAMs polarization to the M1 phenotype. SN/Mn@gHE release SN38 and Mn^2+^ to induce DNA damage and stimulate cGAS-STING activation, respectively. SN/Mn@gHE enhances maturation of DCs and promotes NK cell recruitment to the tumor region and CTL infiltration, resulting in significant antimetastatic and anti-tumor efficacy (114). PEG-Q11T3R4 @CDN inhibits tumor growth and suppresses tumor metastasis through activating the STING pathway in CRC (115). SATE-based prodrugs of dCDNs decreases CT26-Luc tumor growth and eliminates the established tumor through activating STING pathway (116). Tetrahedral DNA nanostructures synergize with MnO_2_(TDN-MnO_2_ complex) exerts anti-tumor response through activating the STING pathway in HCC (117). ISAMn-MOF inhibits tumor growth and metastasis through activating the cGAS-STING pathway in melanoma (119). CDG-NPs enhance the retention and intracellular delivery of CDG in the tumor site and facilitates activation of STING and TME immunogenicity to enhance STING-mediated anti-tumor immunity in melanoma-bearing mice (120).

### 4.4 Chemotherapy agents

Cisplatin inhibits the proliferation of bladder cancer through enhancing accumulation of dsDNA to activate cGAS-STING signal in bladder cancer (123). Paclitaxel, doxorubicin, and cisplatin promote CD8+ T anti-tumor immunity through enhancing induction of cancer cell-autonomous type I interferon via ROS-triggered oxidized mtDNA to activate cGAS-STING in breast cancer (94).

## 5 Conclusions and perspectives

Mounting evidence indicates that cGAS-STING pathway activation plays a vital role in the pathogenesis of diseases, including cancers. Emerging studies have demonstrated that pharmacological activation of the cGAS-STING pathway offers a novel therapeutic opportunities to treat cancers. Many bioactive compounds exert potential therapeutic effects against cancers by activating or inactivating the cGAS-STING pathway. In this review, we first outline the principal core mechanisms of activation for cGAS-STING signaling, then summarize recent research mechanistically connecting cGAS-STING signaling to the pathogenesis of cancers. Finally, we outlined several bioactive compounds serving as potential pharmacological antagonists of the cGAS-STING pathway, delineating their beneficial effects against the phenotypes in cancers. This review spotlights the novel potential of cGAS-STING agonists as novel therapeutic agents against cancers.

However, many questions remain to be answered. First, cGAS-STING signaling is tightly regulated at the level of transcriptional regulation, posttranslational modifications, and epigenetic modifications in various diseases, especially in cancer. An area that merits future study is the interplay between the activation of cGAS-STING and various regulated cell death (RCD) contributory to disease pathogenesis, such as: ferroptosis, autophagy, pyroptosis, etc. These interplay needs to be explored on a per-disease basis. The role played by the cGAS-STING pathway is disease dependant, and places the cGAS-STING pathway as a doubled-edged sword, which may be inhibited or activated to arrive at the desired outcome. cGAS-STING activation may result in pathological conditions in non-cancer diseases. For instance, tumor cells evade cGAS-STING, and activation of this axis offers benefits in specific forms of cancer. Activation of cGAS-STING may kill cancer by overcoming resistance to targeted therapy, conventional chemotherapy, and immunotherapy. It is essential to uncover which genes and proteins regulate cGAS-STING in specific diseases, along with what their initial triggering insults are. Identifying the diverse regulators of ferroptosis in cancers remains a challenge to be resolved. Lastly, most results reported in the literature on the role played by cGAS-STING in diseases are derived from experimental studies, which do not directly related to clinical implications and applications. Many agonists of STING have been tested in clinical trials for cancer immunotherapy (2, 130). So far, these clinical trials have yielded disappointing results, with a general failure of efficacy either in combination with checkpoint blockade or as a monotherapy (131). The reasons underlying these outcomes remain unclear and remains an open conundrum for future investigate on. Cumulatively, we need to conduct more clinical studies to inform the development of practical targeted treatment strategies in the future. Translational potential of agonists of cGAS and STING is our ultimate goals. However, the laboratory insights move into clinical trials have potential barriers. cGAS-STING is highly versatile and context-dependent, and cGAS-STING axis is discovered to respond to a wide range of endogenous nucleic acids implicated in cellular stress and damage, and that its signaling outputs reach far beyond IRF3 activation and cytokine induction, such as ferroptosis, autophagy, senescence, cell death, metabolism regulation, DNA damage response, and RNA replication restriction. The in-depth cGAS-STING interactome and mechanisms of versatile outputs deserve further investigation. Our understanding of the functions and mechanisms of the DNA-sensing pathway cGAS-STING has grown exponentially since the descriptions of intracellular DNA sensing (132, 133), the discovery of STING (28, 34), and the identification of cGAS and cGAMP (22, 23). These remarkable achievements benefit from two important technologies, cryo-EM and CRISPR-Cas9 editing (2). Recent technological advancements such as single-cell RNA sequencing and genetic lineage tracing maybe help to reveal novel cell types and enriched functional properties of existing cell types in different organs that express cGAS-STING.

In summary, despite these considerations, emerging evidence strongly suggests that cGAS-STING pathway induction for cancer is a significant new direction for treating diseases. Direct research on cGAS-STING aligned towards diseases pathogenesis is still needed, but pharmacological agonism of cGAS-STING may be a promising therapeutic approach for cancers.
